# Environmental Epigenetics

**DOI:** 10.1093/eep/dvv002

**Published:** 2015-06-18

**Authors:** Michael K. Skinner

**Affiliations:** Center for Reproductive Biology, School of Biological Sciences, Washington State University, Pullman, WA 99164-4236, USA

With over 350 journals, Oxford University Press is one of the largest publishers of scientific journals. In considering their portfolio recently, they realized they had no specific journals in the rapidly growing area of epigenetics. When they approached me about helping to establish a journal in this area, I considered what other journals had been developed. Several fine journals have been developed in the areas around molecular epigenetics (e.g. *Epigenetics*, *Epigenetics and Chromatin*, and *Epigenomics*) and disease epigenetics (e.g. *Clinical Epigenetics* and *Medical Epigenetics*), with ∼10 in total journals currently focused on epigenetic topics. One of the main areas of epigenetics not currently addressed is environmental epigenetics. Therefore, I agreed to assist Oxford University Press to establish a journal in this area to be called *Environmental Epigenetics* and act as its founding editor-in-chief.

**Figure 1.**
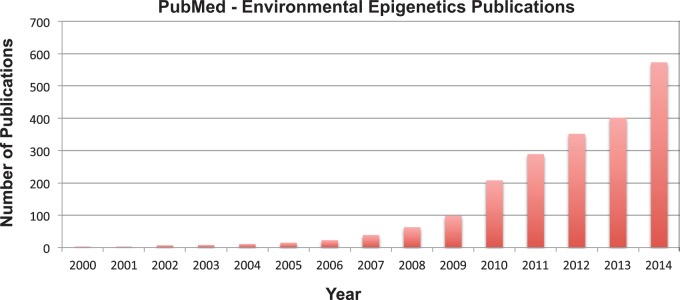
Publication Frequency in Environmental Epigenetics.

The field of epigenetics started in the 1940s with Conrad Waddington, who coined the term, studying environment–gene interactions and non-Mendelian genetic phenomena. Epigenetic molecular markers were first identified in the 1970s with DNA methylation, but it was not until the late 1980s and 1990s when many of the epigenetic processes (DNA methylation, histone modifications, chromatin structure, and non-coding RNA) were identified. To put this in perspective, a search of PubMed using the term “epigenetics” yields ∼12 000 publications, 11 400 (95%) of which were published in the past 5 years. This reflects the dramatic recent growth in the field. Within the area of epigenetics, the largest sub-topic is molecular epigenetics at 40%, then disease epigenetics at over 30% followed by environmental epigenetics at nearly 25% of the literature published. Growth in the area of environmental epigenetics is shown in Fig. 1, based on PubMed information.

Epigenetics provides the molecular conduit between the environment and regulation of genome activity. The majority of environmental factors cannot alter DNA sequence, but most can alter genome function and biology. The area of environmental epigenetics involves a large number of distinct topics. One such topic is toxicology, due to the role of epigenetics in the actions of a wide variety of toxicants and environmental compounds. Another is disease, as that is influenced by the environment and epigenetic mechanisms. A growing number of studies also suggest a role for environmental epigenetics in evolutionary biology. Therefore, the scope of *Environmental Epigenetics* is broad and includes environmental impacts on epigenetics at both a molecular and a physiological level involving all living organisms. This covers areas ranging from evolution, ecology, and population epigenetics to medicine, disease etiology, and the developmental origins of disease. How the environment impacts the molecular mechanisms and processes involved in epigenetics and genetics is included, whether this impacts normal cell and developmental biology or abnormal physiology and toxicology.

*Environmental Epigenetics* will be a completely open access online journal. A streamlined submission, review, and publication process has been established, as is normal for Oxford University Press online journals. A list of suggested reviewers is required of authors and a minimum of three reviews will be sought. Once two reviews have been received, a decision will be made. This will assure a fast turn around in the review process. The journal will work to review all submitted manuscripts. The review will assess whether the study is sound and has a good experimental design and good data interpretation. Innovation and novelty will be considered, but the journal feels the readership is best suited to judge this rather than the reviewers or editors.

A stellar Editorial Board has been assembled that will facilitate the management of the reviewing process. A Consulting Editorial Board will also advise and assist in reviews when needed, and an Editorial Review Board has been established that will assist in the reviews. A list of the Editors can be found at the *Environmental Epigenetics* web site www.enviro- epigenetics.org and information can be obtained at envepi.editorialoffice@oup.com

We encourage you to submit your papers to *Environmental Epigenetics* and I am confident that the journal will provide the optimal venue for the rapidly developing field of epigenetics.

**Table 1.** Editorial Boards and Members

**Editorial Board**	
Baccarelli, Andrea	Harvard University, USA
Bales, Karen	University of California, Davis, USA
Blumberg, Bruce	University of California, Irvine, USA
Bonduriansky, Russell	University of New South Wales, Australia
Chang, Howard	Stanford University, USA
Cheng, Xiaodong	Emory University School of Medicine, USA
Dolinoy, Dana	University of Michigan, USA
Hanson, Mark	University of South Hampton, United Kingdom
Jirtle, Randy	North Carolina State University, USA
Kelly, William	Emory University, USA
LaSalle, Janine	University of California, Davis, USA
Mann, Melissa	University of Western Ontario, Canada
Mansuy, Isabelle	University/ETH Zürich, Switzerland
McCarrey, John	University of Texas at San Antonio, USA
Meissner, Alexander	Harvard University, USA
Metz, Gerlinde A.S.	University of Lethbridge, Canada
Osteen, Kevin	Vanderbilt University School of Medicine, USA
Petronis, Art	University of Toronto, Canada
Ruden, Douglas	Wayne State university, USA
Shioda, Toshihiro	Harvard Medical School, USA
Spencer, Hamish	University of Otago, New Zealand
Sung, Sibum	The University of Texas at Austin, USA
Surani, Azim	Cambridge University, United Kingdom
Szyf, Moshe	McGill University, Canada
Waterland, Robert	Baylor College of Medicine, USA
Weitzman, Jonathan	Université Paris Diderot, France
Yan, Wei	University of Nevada Reno, USA
**Consulting Board**	
Gluckman, Peter	The University of Auckland New Zealand, New Zealand
Guillette, Lou	Medical University of South Carolina, USA
Jablonka, Eva	Cohn Institute, Tel Aviv University, Israel
Jégou, Bernard	INSERM University, France
Peterson, Richard	University of Wisconsin, USA
Rando, Oliver	University of Massachusetts Medical School, USA
Ressler, Kerry	Emory University, USA
Swanson, Penny	Northwest Fisheries Science Center, NOAA- Fisheries, USA
Tonellato, Peter	University of Wisconsin Milwaukee, USA
vom Saal, Frederick	University of Missouri-Columbia, USA
**Editorial Review Board**	
Bhandari, Ramji	University of Missouri, USA
Breton, Carrie	University Southern California, USA
Burghardt, Kyle J.	Wayne State University, USA
Burris, Heather	Harvard University, USA
Chen, Jia	Mount Sinai, USA
Colacino, Justin	University of Michigan, USA
Colicino, Elena	Harvard University, USA
Cropley, Jennifer	Victor Chang Cardiac Research Institute, Australia
Davie, James	Manitoba Institute of Child Health, Canada
Dearden, Peter K.	University of Otago, New Zealand
Dias, Brian G.	Emory University, USA
Dinger, Marcel	Garvan Institute of Medical Research, UNSW, Australia
Faulk, Christopher	University of Michigan, USA
Fry, Rebecca	University of North Carolina, USA
Golding, Michael	Texas A&M University, USA
Goodrich, Jaclyn	University of Michigan, USA
Greer, Eric Lieberman	Harvard Medical School, USA
Guerrero-Bosagna, Carlos	Linköping University, Sweden
Hostetler, Caroline	Oregon Health & Science University, USA
Houghton, Franchesca	University of Southampton, United Kingdom
Hoyo, Cathrine	North Carolina State University, USA
Iderabdullah, Folami	University of North Carolina, USA
Just, Allan	Harvard University, USA
Kelsey, Gavin	The Babraham Institute, Cambridge, United Kingdom
Kile, Molly	Oregon State University, USA
Kimmins, Sarah	McGill University, Montreal, Canada
Kinnally, Erin	University of Southern California at Davis, USA
Kotaja, Noora	Institute of Biomedicine, Finland
Kovalchuk, Igor	University of Lethbridge, Canada
Kovalchuk,Olga	University of Lethbridge, Canada
Kramer, Jamie	University of Western Ontario, Canada
Laiosa, Michael	University of Wisconsin, USA
LaMerrill, Michelle	University of California at Davis, USA
Marsit, Carmen	Dartmouth University, USA
McCullough, Shaun D.	U.S. EPA, Chapel Hill, NC, USA
Medici, Valentina	University of California, Davis, USA
Meyer, Ralph G.	Utah State University, USA
Miska, Eric	Gurdon Institute, Cambridge, United Kingdom
Morison, Ian	University of Otago, Dunedin, New Zealand
Murphy, Susan K.	Duke University, USA
Nagel, Susan C.	University of Missouri-Columbia, USA
Nakagawa, Shinichi	University of New South Wales, Sydney, Australia
Ng, Jane	University of Calgary, Canada
Nilsson, Eric	Washington State University, USA
Olson, David	University of Alberta, Canada
Öst, Anita	Linköping University, Sweden
Owens, Julie	University of Adelaide, Australia
Rassoulzadegan, Minoo	Inserm University, France
Rivera, Rocio	University of Missouri, USA
Robert, Claude	Laval Université, France
Roth, Tania	University of Delaware, USA
Saffery, Richard	Murdoch Childrens Research Institute, Melbourne, Australia
Saha, Ramendra	University of California, Merced, USA
Schmidt, Rebecca	University of California Davis MIND Institute, USA
Sharma, Abhay	CSIR-Institute of Genomics and Integrative Biology, New Delhi
Skaar, David	North Carolina State University, USA
Stolzenberg, Danielle	University of California Davis, USA
Sultan, Sonia	Wesleyan University, USA
Suter, Catherine	Victor Chang Cardiac research Institute, Australia
Trasler, Jacquetta	McGill University, Canada
Watson, Erica	University of Cambridge, United Kingdom
Youngson, Neil	The University of New South Wales, Australia
Zama, Aparna	Rutgers University, USA
Zeh, David W.	University of Nevada, Reno, USA
