# Supplementary figures and images for: Environmental Epigenetics
Source: Environ Epigenet. 2015 Jun 18;1(1):dvv002. doi: 10.1093/eep/dvv002 (PMC5804580; doi:10.1093/eep/dvv002)

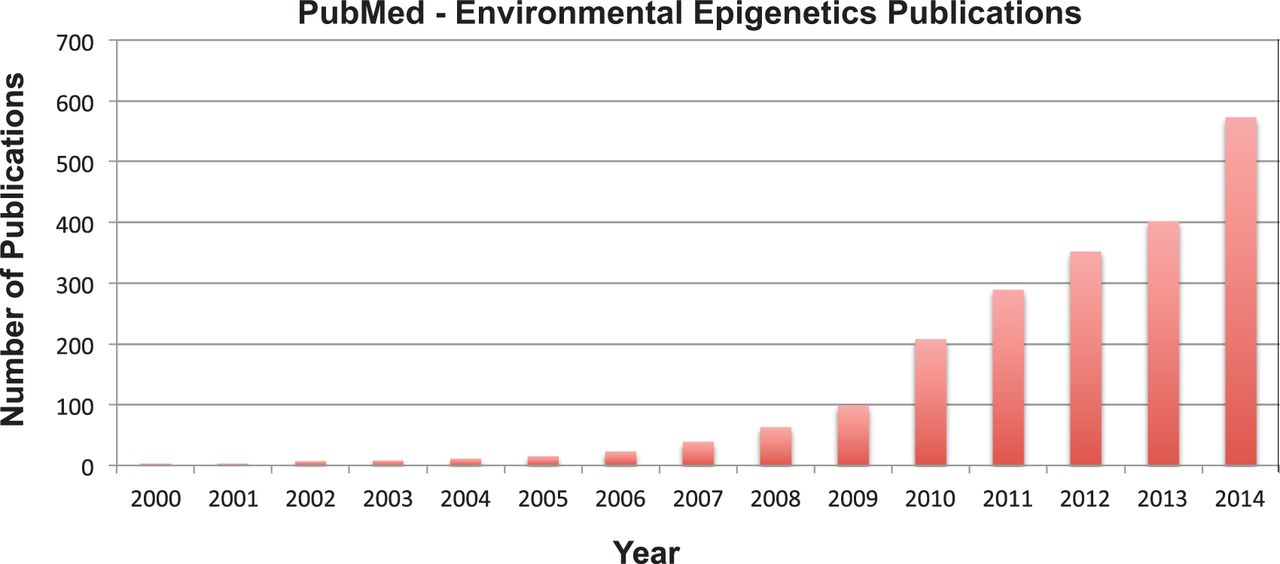

Supplement: Supplementary file 1 [file dvv002-f1.ppt]
